# Nasopharyngeal Microbiota in Children With Invasive Pneumococcal Disease: Identification of Bacteria With Potential Disease-Promoting and Protective Effects

**DOI:** 10.3389/fmicb.2019.00011

**Published:** 2019-01-28

**Authors:** Anny Camelo-Castillo, Desirée Henares, Pedro Brotons, Antonio Galiana, Juan Carlos Rodríguez, Alex Mira, Carmen Muñoz-Almagro

**Affiliations:** ^1^Institut de Recerca Sant Joan de Déu, Hospital Sant Joan de Déu, Barcelona, Spain; ^2^Network of Epidemiology and Public Health, CIBERESP, Barcelona, Spain; ^3^Department of Microbiology, Hospital de Alicante, Alicante, Spain; ^4^Center for Advanced Research in Public Health, FISABIO Foundation, Valencia, Spain; ^5^School of Medicine, Universitat Internacional de Catalunya, Barcelona, Spain

**Keywords:** nasopharyngeal microbiota, children, invasive pneumococcal disease, *Streptococcus pneumoniae*, *Dolosigranulum*

## Abstract

**Background and Aims:** The risk of suffering from some infectious diseases can be related to specific microbiota profiles. Specifically, the nasopharyngeal microbiota could play a role as a risk or protective factor in the development of invasive disease caused by *S. pneumoniae*.

**Methodology:** We analyzed the nasopharyngeal microbiota of children with invasive pneumococcal disease (IPD) and that of healthy controls matched by age, sex, and seasonality from Catalonia, Spain. Epidemiological, microbiological and clinical variables were considered to compare microbiota profiles, analyzed by sequencing the V1–V4 region of the 16S rRNA gene.

**Results:** Twenty-eight children with IPD (median age 43 months) and 28 controls (42.6 months) were included in the study. IPD children presented a significantly higher bacterial diversity and richness (*p* < 0.001). Principal coordinate analysis revealed three different microbiota profiles: microbiota A, dominated by the genus *Dolosigranulum* (44.3%); Microbiota B, mostly represented by *Streptococcus* (36.9%) and *Staphylococcus* (21.3%) and a high diversity of anaerobic genera including *Veillonella, Prevotella* and *Porphyromonas*; and Microbiota C, mainly containing *Haemophilus* (52.1%) and *Moraxella* (31.4%). The only explanatory factor for the three microbiotas was the classification of children into disease or healthy controls (*p* = 0.006). A significant negative correlation was found between *Dolosigranulum* vs. *Streptococcus* (*p* = 0.029), suggesting a potential antagonistic effect against pneumococcal pathogens.

**Conclusions:** The higher bacterial diversity and richness in children with IPD could suggest an impaired immune response. This lack of immune competence could be aggravated by breastfeeding <6 months and by the presence of keystone pathogens such as *Porphyromonas*, a bacterium which has been shown to be able to manipulate the immune response, and that could favor the overgrowth of many proteolytic anaerobic organisms giving rise to a dramatic dysbiosis. From an applied viewpoint, we found suggestive microbiota profiles associated to IPD or asymptomatic colonization that could be used as disease biomarkers or to pave the way for characterizing health-associated inhabitants of the respiratory tract. The identification of beneficial bacteria could be useful to prevent pneumococcal infections by integrating those microorganisms in a probiotic formula. The present study suggests not only respiratory tract samples, but also breast milk, as a potential source of those beneficial bacteria.

## Introduction

The human microbiota communities and their genes are an intriguing ecosystem that play an essential role for human body functions including food digestion, nutrition, regulation of human metabolism, and regulation of immune defense against infections, among others (Zhu et al., 2010). The majority of microorganisms of the human ecosystem act as symbionts that co-evolve and co-adapt with their human host according to their diet, life-style, human genetic characteristics, immune modulation, or environment parameters (Dethlefsen et al., 2007). Particularly in samples from the respiratory tract, the variability in bacterial composition between individuals has been described to be so high that a core microbiome could not be defined at the species level (Bogaert et al., 2011). Despite this inter-individual variability, the bacterial composition of each person has been proposed to fall within discrete categories where some bacterial taxa dominate the community. The presence of these structured microbial consortia was first described in the gut, where three groups or “enterotypes” were found, dominated by *Bacteroides, Prevotella*, or *Ruminococcus* (Arumugam et al., 2011). However, the notion of enterotypes is currently under debate, and it is still not clear whether enterotypes can be generalized to the general population, or whether microbiota follows in fact a gradient in taxonomic composition (Jeffery et al., 2012). In children between 1 and 3 months of age, for instance, the hypopharynx was found to contain four or five community types, and these “pneumotypes” followed specific trajectories during the child's development (Mortensen et al., 2016). In adults, bronchoalveolar lavage samples showed two distinct pneumotypes, one of which was enriched with oral microorganisms (particularly *Prevotella* and *Veillonella*) and appeared to be associated with higher inflammatory markers (Segal et al., 2013). Thus, studying the bacterial community structures in the respiratory tract could be important to understand susceptibility to Chronic Obstructive Pulmonary Disease (COPD) or to pulmonary infections like pneumococcal pneumonia or, more extensively, Invasive Pneumococcal Disease (IPD).

It has been proposed that the degree of stability of the microbiota depends partly on the first colonization by keystone species in the first days of life (Relman, 2012). Natural feed of humans in their early days is breastfeeding, so human milk is expected to be a major supplier of keystone species for human microbiota development, including the respiratory tract, given the high microbial diversity in colostrum, transition, and mature milk (Hunt et al., 2011; Cabrera-Rubio et al., 2012). In addition, breast milk provides macronutrients, micronutrients and bioactive molecules that protect against infections and inflammation and are key factors for human growth and development (Ballard and Morrow, 2013). Another source of resilience to infection is provided by the antagonistic interactions between the local microbiota and the invasive species. For instance, the nasal passage inhabitant *Corynebacterium accolens* appears to be over-represented in children without *Streptococcus pneumoniae* nasal colonization and has been shown to release antipneumococcal free fatty acids from human skin surface triacylglycerols (Bomar et al., 2016). Other commensal bacteria produce antimicrobial peptides that have been shown to inhibit oral pathogens (López-López et al., 2017) and prevent pharynx infections in children (Di Pierro et al., 2014). Thus, understanding microbiota composition and structure in respiratory samples will be instrumental for establishing disease risk and for the development of probiotics that could be used to colonize the respiratory tract and contribute to the prevention of infections (Tagg and Dierksen, 2003; Medina et al., 2008).

Our hypothesis is that the nasopharyngeal microbiota plays a role as a risk or protective factor in the development of invasive disease caused by *S. pneumoniae*. To test this hypothesis, the objective of the present study was to characterize the nasopharyngeal microbiota profiles in two groups of children: (i) children with invasive pneumococcal disease (IPD), considered as a case group whose nasopharyngeal microbiota was suffering an important disturbance; and (ii) a matched control group of healthy children representative of a healthy nasopharyngeal niche. A series of epidemiological, microbiological, and clinical variables related with major risk of developing IPD were considered to compare microbiota profiles in the two groups. For robust characterization of bacterial composition, a long-fragment 16S sequencing approach was used, in which a 780 bp region of the gene was amplified and sequenced in order to improve taxonomic assignment.

## Materials and Methods

### Study Design

This was an observational prospective study including all children and adolescents (<18 years) with IPD admitted to 5 hospitals in Catalonia, Spain: Hospital Sant Joan de Deu, Hospital de Nens, Hospital de Mataro, Hospital de Vic and Hospital de Calella (HC), during the period January 2014 to March 2015. One healthy control that was treated at Sant Joan de Deu hospital for minor surgery (i.e., phimosis or minor dermatologic surgery) was selected for each case. Cases and controls were matched by age, sex, and seasonality, given previous evidence of seasonal influence on the microbiota composition of respiratory samples (Bogaert et al., 2011). Children whose parents or guardians did not sign the informed consent were excluded of the study. The study was performed following the guidelines of the Ethics Committee of Hospital Sant Joan de Deu which approved the study. All information collected has been treated confidentially and in accordance with applicable laws on personal data.

### IPD Definition

IPD was defined as the presence of clinical findings of infection (which were used for classification of the disease) together with isolation of *Streptococcus pneumoniae* and/or DNA detection of autolysin (*LytA*) gene and an additional capsular gene of *S. pneumoniae* by Real-Time PCR in plasma, cerebrospinal fluid or pleural fluid. Clinical diagnoses were mutually exclusive.

### Data Collection

Relevant clinical, epidemiological, immunological, and microbiological variables were collected for each subject including delivery mode, duration of breastfeeding (mixed or exclusive), fulfillment of breastfeeding world health recommendations (minimum duration of 6 months) exposure to crowding, pneumococcal vaccination status, parents' smoking habits, and previous occurrence of respiratory infections. Among microbiological variables we assessed carriers of 13-valent pneumococcal conjugate vaccine (PCV13) serotypes, invasiveness potential of nasopharyngeal *S. pneumoniae* serotypes and co-colonization with respiratory viruses. Serotypes were classified according to the studies of Brueggemann et al. (2003), Sleeman et al. (2006), and del Amo et al. (2014). Serotypes 1, 3, 4, 5, 7F, 8, 9A, 9V, 12F, 14, 18C, and 19A were considered to have a high-attack rate or highly-invasive serotypes whereas the remainder were considered as low-attack rate or non-highly-invasive or opportunistic serotypes. In addition, we recorded data of clinical diagnosis, presence of underlying disease, previous antibiotic intake, white blood cell count, C-reactive protein level, occurrence of complications, length of hospitalization, provision of intensive care, and course of disease for the cases.

### Sample Collection and Laboratory Analyses

Nasopharyngeal aspirate samples collected in sterile plastic tubes from cases and controls and eluted with 0.5 ml of phosphate buffered saline (PBS) were extracted and stored at −80°C until further genomic/microbial analysis. Whenever samples were needed for the sole purposes of the study they were taken by experienced nurses of the Hospital Sant Joan de Deu Clinical Trials Unit.

### Microbiological Methods

#### *S. pneumoniae* Detection and Characterization

The definition of Invasive Pneumococcal Disease (IPD) is the presence of clinical findings of infection together with isolation of *Streptococcus pneumoniae* by culture and/or DNA detection of *S. pneumoniae* by Real-Time PCR in plasma, cerebrospinal fluid or any other sterile fluid. The microbiological confirmation of the patients is based on one technique, the other or both together. All pneumococcal isolates were identified by standard microbiological methods, including the optochin sensitivity test and an antigenic test targeting the capsular polysaccharide (Slidex pneumo-kit, bioMérieux, Marcy-l'Etolie, France). DNA detection of pneumococcal *LytA* gene was performed by a duplex real-time PCR as previously reported (Selva et al., 2014; del Amo et al., 2015). This duplex real-time PCR also included as target the *Rnase P* human single-copy gene to detect and measure the number of human cells (Brotons et al., 2017), using primers and probes recommended by CDC (Meningitis, 2011). Absolute quantification of RNAseP using PCR can be used to estimate the number of human cells present in the clinical samples. The measure of Human DNA load was then used to normalize by potential differences in sample amount and avoid potential bias between samples. In addition, DNA was measured prior to PCR and concentrations adjusted to minimize amplification bias between samples. Capsular typing was carried out by a molecular technique based on automated fluorescent fragment analysis which allows differentiation of 40 serotypes (Selva et al., 2014). Quellung reaction performed at the National Center for Microbiology (Majadahonda, Madrid) was used to complete serotyping in invasive strains isolated by culture.

#### Viral Respiratory Study in Nasopharyngeal Samples

All nasopharyngeal samples were tested by AnyplexTM II RV16 Detection v1.1. (Seegene, Seoul, Korea). This multiplex Real-Time PCR assay performs simultaneous amplification, detection and differentiation of DNA/RNA of Adenovirus (AdV), Influenza A virus (FluA), Influenza B virus (FluB), Parainfluenza virus 1 (PIV1), Parainfluenza virus 2 (PIV2), Parainfluenza virus 3 (PIV3), Parainfluenza virus 4 (PIV4) Rhinovirus A/B/C (HRV), Respiratory syncytial virus A (RSV A), Respiratory syncytial virus B (RSV B), Bocavirus 1/2/3/4 (HBoV), Metapneumovirus (MPV), Coronavirus 229E (CoV 229E), Coronavirus NL63 (CoV NL63), Coronavirus OC43 (CoV OC43), Enterovirus (HEV), and an internal control in two PCR sets.

### Microbiota Analysis

DNA extraction of nasopharyngeal aspirates was performed by Nuclisens® EasyMag® according to manufacturer instructions (bioMérieux, Marcy-l'Etolie, France). This is an automated system for total nucleic acid extraction based in magnetic silica particles. After extracting the DNA, its quality and quantity was measured with Nanodrop (Thermo Fisher Scientific, Massachusetts, USA) and only samples with a 260/280 absorbance ratio between 1.8 and 2 were processed for Next Generation Sequencing (NGS).

NGS libraries were created with 100 ng of total DNA for each sample, to which a unique multiplex identifier (MID) was assigned. PCR amplification of the 16S rRNA gene was performed using high fidelity Extensor Long Range PCR Enzyme (Thermo Fisher Scientific, Massachusetts, USA), with the degenerate universal bacterial primers of 16S rRNA gene 8F (5′-CAGAGTTTGATCMTGGCTCAG-3′) and 785R (5′-GGCCVGGGTATCTAATCC-3′) (Simón-Soro et al., 2014) and the following cycling conditions to minimize amplification biases (Simón-Soro et al., 2014): 2 min at 94°C, followed by 30 cycles of 10s at 94°C, 30s at 52°C, 90s at 68°C, and a final step of 7 min at 68°C. These primers amplified the variable regions 1 to 4 (V1–V4) of 16S rRNA gene with an expected size of 780 pb. The PCR products were first purified using a Minielute PCR purification Kit (Qiagen, Venlo, The Netherlands) and then using Agencourt AMPure beads (Beckman coulter, Munich, Germany). Finally, the amplicons were pyrosequenced using a 454 GS FLX Titanium chemistry (Roche, Basel, Switzerland) with Lib-L type microspheres, pooling 20 samples per 1/8 of a pyrosequencing plate.

Negative controls (PBS) were extracted by the same method of the studied samples (Easymag) and 16S rRNA gene and Human *Rnase P* gene were analyzed by PCR, producing no amplification. DNA concentrations for negative controls were also below the quality control thresholds required at Macrogen Inc. (Republic of South Korea). In addition, a negative control (water) was included in each sequencing run to discard contamination during library preparation, producing no results.

### Bioinformatic Analysis and OTU Assignment

Raw sequences were analyzed using “Quantitative Insights into Microbial Ecology” (QIIME) v1.7.0 software (Caporaso et al., 2010) and were separated using the 8-bp “barcodes” assigned to each sample. Chimeric sequences were eliminated by ChimeraSlayer program (Haas et al., 2011). An end-trimming quality filtering was performed by removing 50-bp windows with quality values <25 using Prinseq. Only reads >500 bp were included for taxonomic classification. Sequences with differences in the primer binding region or with more than four ambiguities in homopolymeric regions were excluded from the analysis. The resulting high quality sequences of the 16S rRNA gene were classified using the RDP database (Ribosomal Database Project) (Cole et al., 2009) with a bootstrap cutoff of 80%. Samples were classified in Operational Taxonomic Units (OTUs) at 97% sequence identity as the standard species-level boundary (Yarza et al., 2008). Only the OTUs representing over 0.1% of the total sequences of each sample were considered for further statistical analysis, as low-frequency reads, including singletons, are more likely to represent sequencing errors, contaminants, or transient organisms without a biological role at the niche under study (de la Cuesta-Zuluaga and Escobar, 2016). Bacterial taxonomic composition was determined for each sample and means for each group (cases and controls) were calculated.

### Estimation of Microbial Diversity and Statistical Analysis

Data analysis was performed using QIIME 1.7.0 software. Rarefaction curves (Mean ± S.E.) were calculated by including 1,000 randomly selected reads per sample. Alpha diversity indexes were calculated from rarefied samples (1,000 sequences per sample), using the Shannon index (Shannon, 1948) for diversity and the Chao1 index (Chao, 1984) for richness. Beta diversity was also calculated with the rarefied 1,000 reads per sample using weighted and unweighted UniFrac (Lozupone et al., 2006) distance matrices, and the principal coordinate analysis (PCoA) generated 2D and 3D plots for all mapping fields. In addition, we clustered samples using UPGMA (Unweighted Pair Group Method with Arithmetic mean, also known as average linkage) (Michener and Sokal, 1957). Two-way comparisons in bacterial composition using the UniFrac metric (Lozupone et al. 2006) were used to measure whether the microbial communities in the microbiota types were significantly different. A tree with 1,000 reads from each sample was obtained, and microbiota types were considered significantly different if the UniFrac distance value for the tree was larger than expected if the sequences were randomly distributed. A thousand permutations were performed to obtain a *P*-value, using Bonferroni corrections for multiple comparisons. In addition, we also performed Constrained Correspondence Analysis (CCA), which is a statistic tool which emphasizes variation, and tests whether the factor provided (the microbiota type) can explain part of the total variability. This analysis was performed by the R software ade4 package (Dray and Dufour, 2007) using the Chi-squared distances-based function CCA. Adonis tests were done with the R library “vegan” (Oksanen et al., 2018) to determine corrected *p*-values.

Microbial community comparisons and statistical analysis were performed using the statistical software R v2.15.2 with the packages for Community Ecology (vegan), Euclidean Methods in Environmental Sciences (ade4), The Bee Swarm Plot (beeswarm) and gplots packages (http://R-project.org), R (The R Foundation for Statistical Computing).

We used LEfSe (Linear discriminant analysis Effect Size) with default parameters (Segata et al., 2011), available in the Galaxy Web Server toolkit to determine significant differences (alpha value = 0.05) in the proportions of bacterial genera between cases and controls groups. LEfSe uses the non-parametric factorial Kruskal-Wallis (KW) sum-rank test to detect features with significant differential abundance with respect to the class of interest.

Normality of the data was analyzed with the Shapiro-Wilk test. Continuous data with normal distributions were described using mean and Standard Deviation (SD). In case of non-normal distribution, median and interquartile range (IQR) were used. Significance (*p*-values) of continuous and normally distributed data was determined using Student's *t*-test. When data did not follow a normal distribution, Wilcoxon signed-rank test was used. Significance of categorical data was established by using Chi-square test. When Chi-square expected frequency was equal or less than five, Fisher's exact test was applied. When variables followed a normal distribution, the ANOVA test was used to determine if there were statistically significant differences in mean values between the microbiota groups. If distribution was not normal, we used the non-parametric Kruskal-Wallis test. Multiple testing correction was performed when needed.

### Ethics Statement

The study was performed following the guidelines of the Ethics Committee of Hospital Sant Joan de Deu which approved the study (project approval code PIC-70-15). All information collected has been treated confidentially and in accordance with applicable laws on personal data. Written consent was obtained from parent or legal guardians of children included in the study.

## Results

### Epidemiological and Clinical Features of Patients

During the study period a total of 53 patients were diagnosed of IPD in Hospital Sant Joan de Deu (*n* = 38), Hospital de Nens (*n* = 6), Hospital de Mataro (*n* = 5), Hospital de Vic (*n* = 2), and Hospital de Calella (*n* = 2). Fourty-two of them (79.2%) accepted to participate in the study under informed consent. Twenty-eight (52.8%) controls were available for matching with cases so the study finally comprised 56 subjects. No significant differences of total human DNA load was found between samples of cases and controls (Log_10_ 3.6 vs. 3.2 gene copies/reaction, *p* = 0.1), indicating that both types of samples did not differ in the amount of material.

The median age of the 28 cases was 43.0 months (IQR 20.8–60.2 months) and 16 (57.1%) were male. The clinical diagnoses of these patients were 19 pneumonia (including 10 complicated pneumonia with empyema and 3 necrotizing pneumonia), 7 bacteremia, 1 meningitis, and 1 fulminant sepsis. Invasive samples collected for diagnosing IPD included blood (*n* = 19, 67.8%), pleural fluid (*n* = 5, 17.9%), blood and pleural fluid (*n* = 3, 10.7%), and cerebrospinal fluid (*n* = 1, 3.6%). Detection of *S. pneumoniae* in invasive samples was performed by PCR (*n* = 14, 50.0%), by culture (*n* = 12, 42.9%), and both by PCR and culture (*n* = 2, 7.1%). Median white blood cell count of 17,515/L and median C-reactive protein levels of 154.7 mg/L indicated inflammatory activity. An underlying disease was reported in 3 cases with chronic pulmonary disease, neuroblastoma, and splenic dysfunction, respectively. Median length of hospitalization was 7 days (IQR 4–8 days). Five cases (17.9%) received intensive care, 4 (14.3%) suffered sequelae, and one child died.

Pneumococcal nasopharyngeal carriage was observed in 18 (64.3%) cases and 16 (57.1%) controls. A total of 11 PCV13 serotypes were identified among carriers in the case group, specifically serotype 1 (*n* = 2), serotype 3 (*n* = 3), serotype 14 (*n* = 2), serotype 19A (*n* = 3), and serotype 19F (*n* = 1). In contrast, only 2 PCV13 serotypes, classified as serotype 3, were found in control carriers. Nasopharyngeal samples were taken from cases after a mean of 92 h of fever (IQR 48–408 h) and all of them except two (one patient with necrotizing pneumonia and another one with fatal fulminant sepsis and viral respiratory coinfection), were exposed to beta-lactamic antibiotic treatment, during a mean period of 4 days. No patients were exposed to macrolide treatment or other class of antibiotics. Table 1 shows epidemiological and microbiological variables for cases and controls. Proportions of coinfection with respiratory virus and pneumococcal nasopharyngeal carriage, were higher in cases compared to controls, but the differences between groups did not reach statistical significance. There was a significantly higher proportion of PCV13 pneumococcal serotype carriers in cases compared to controls (61.1 vs. 12.5%, *p* = 0.004) as well as significantly higher number of high-attack rate serotypes (55.5 vs. 12.5%, *p* = 0.009). A trend for significance was observed for breastfeeding through 6 months (the minimum time recommended by World Health Organization) in cases (39.1 vs. 68.4%, *p* = 0.06).

**Table 1:** Epidemiological and microbiological characteristics of study groups.

**Variable**	**Cases (*n* = 28)**	**Controls (*n* = 28)**	***p*-value**
**EPIDEMIOLOGICAL VARIABLES**			
Age, months (Md, IQR)	43.0 (20.8–60.2)	42.6 (31.6–58.9)	0.72
Gender, male	16 (57.1)	16 (57.1)	1.00
Influenza season	12 (42.9)	14 (50.0)	0.59
Race, Caucasian	23 (82.1)	25 (89.3)	0.71
Birth weight, gr (Mean, SD)	3,143 (626)	3,298 (579)	0.34
Gestational age, weeks (Mean, SD)	38.5 (2.3)	38.8 (2.0)	0.67
Delivery type, C–section^†^ (cases: *n* = 25; controls: *n* = 26)	7 (28.0)	10 (38.5)	0.43
Breastfeeding	23 (82.1)	19 (67.9)	0.22
Breastfeeding duration ≥6 months^¶^ ^†^ (cases: *n* = 23; controls: *n* = 19)	9 (39.1)	13 (68.4)	0.06
Breastfeeding duration, months (Md, IQR)^†^ (cases: *n* = 23; controls: 19)	4 (1–7)	6 (4–10)	0.19
Day-care attendance	25 (89.3)	23 (82.1)	0.45
Shared bedroom^†^ (cases: *n* = 21; controls: 27)	13 (61.9)	12 (44.4)	0.23
Parental smoking^†^ (cases: *n* = 26)	11 (42.3)	13 (46.4)	0.76
Vaccinated against influenza	1 (3.6)	0 (0.0)	1.00
Vaccinated against pneumococcal disease	17 (60.7)	19 (67.9)	0.58
PCV13 vaccinated	14 (82.4)	17 (89.5)	0.42
Respiratory infection in the previous 30 days^†^ (Controls: *n* = 27)	13 (46.4)	13 (48.1)	0.90
**MICROBIOLOGICAL VARIABLES**			
Sp detection in NP sample by PCR	18 (64.3)	16 (57.1)	0.58
High-attack rate Sp serotype in NP sample	10 (55.5)	2 (12.5)	**0.009**
PCV13 serotype coverage in NP sample	11 (61.1)	2 (12.5)	**0.004**
Viral detection in NP sample by PCR	17 (60.7)	14 (50.0)	0.42
Rinovirus	10 (35.7)	10 (35.7)	1.00
Adenovirus	4 (14.3)	4 (14.3)	1.00
Parainfluenza virus	2 (7.1)	1 (3.6)	1.00
Respiratory siyncytial virus	2 (7.1)	0 (0.0)	0.49
Coronavirus	1 (3.6)	2 (7.1)	1.00
Enterovirus	1 (3.6)	0 (0.0)	1.00
Bocavirus	1 (3.6)	0 (0.0)	1.00
Influenza A virus	2 (7.1)	0 (0.0)	0.49
Influenza B virus	0 (0.0)	1 (3.6)	1.00
Multiple respiratory viral infection	5 (29.4)	4 (28.6)	1.00

*Results expressed as counts (%) unless stated otherwise*.

*Md, median; IQR, interquartile range; SD, standard deviation; PCV13, 13-valent pneumococcal conjugate vaccine; PICU, pediatric intensive care unit*.

† *missing values*.

¶ *according to WHO recommendations*.

*The bold values means statistically significant associations with p-values < 0.05*.

### High Bacterial Diversity in IPD Patients

A total of 284.558 16S rRNA sequence reads were obtained. After sequence length and quality filtering, we obtained 201.888 reads >500 bp (500–780 bp), with an average of 2.549 reads of the 16S rRNA gene per sample (range between 1,085 and 29,370 reads).

**Figure 1:**
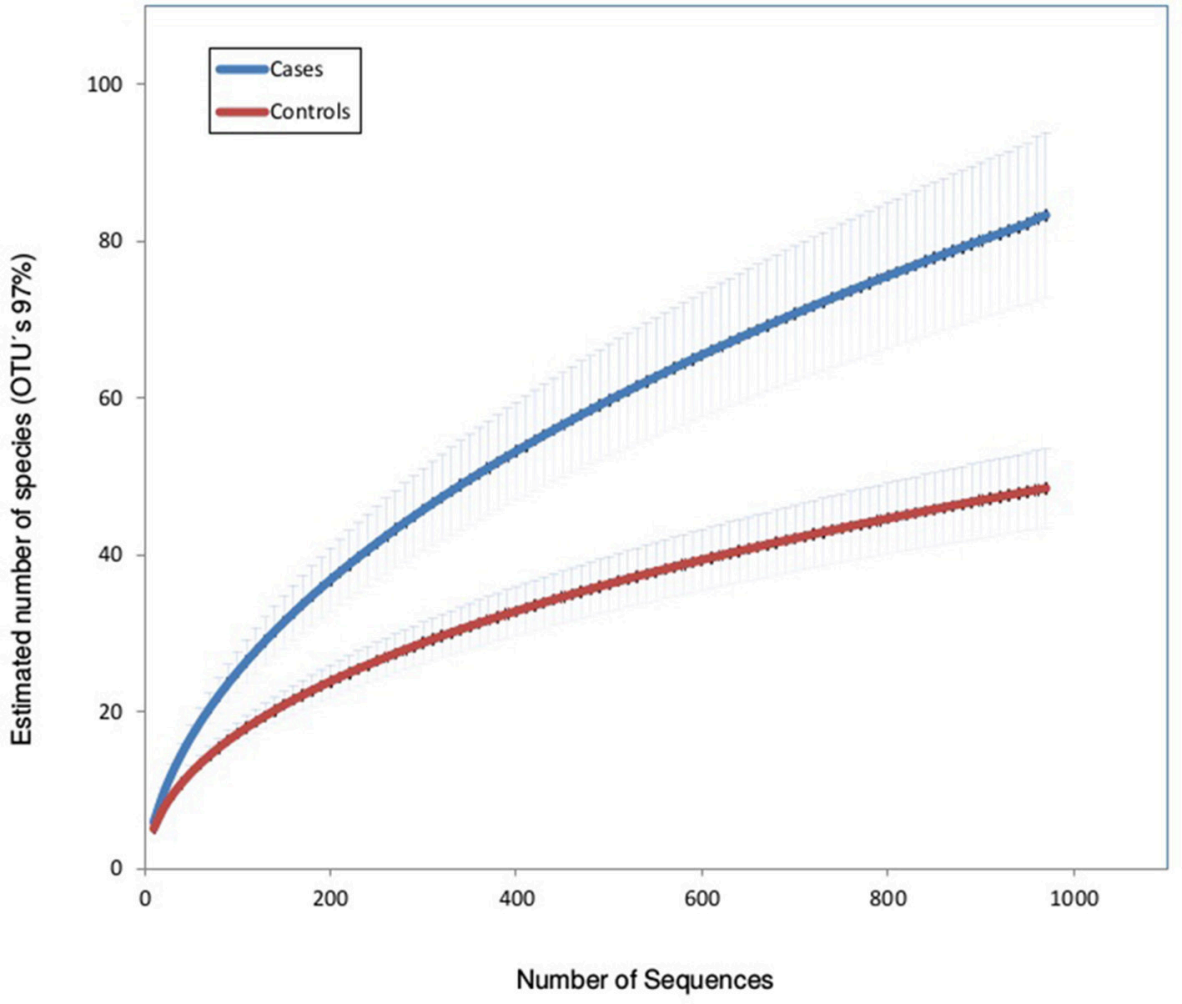
Rarefaction curves of nasopharyngeal samples in patients with invasive pneumococcal disease (cases) and healthy children (controls). The horizontal axis shows the number of reads (sequencing effort) obtained by pyrosequencing the 16S rRNA gene. The vertical axis shows the number (mean ± S.E.) of operational taxonomic units (OTUs) at a level of 97% (estimated mean number of bacterial species).

Rarefaction curves (using OTUs at 97% sequence identity) showed that mean bacterial richness had higher values in children with IPD compared with healthy patients (Figure 1). Individual rarefaction curves with 1,000 reads show that most bacteria present were detected by this level of coverage and show a shift toward higher OTU richness in children with IPD (Supplementary Figure 1). Species richness (Chao1 Index) was significantly higher in cases (mean: 79, 95% confidence interval: 57.4–100.58) compared to healthy controls (mean: 47.8, 95% CI: 37.5–58.2, *p* < 0.0001). The Shannon diversity index was also significantly higher in cases (mean: 1.21, 95% CI: 0.94–1.48) vs. healthy controls (mean: 0.77, 95% CI: 0.62–0.92, *p* < 0.0001).

The taxonomic assignment of the 16S rRNA reads revealed that the most common phyla in IPD patients were *Firmicutes* (50.9% of the total number of reads), *Proteobacteria* (41.4%), *Bacteroidetes* (5.0%), *Actinobacteria* (2.2%), and a lower proportion of *Fusobacteria*. Within the control group, the most common phyla were *Proteobacteria* (66.0% of the total number of reads), *Firmicutes* (33.6%), *Bacteroidetes* (0.2%), and *Actinobacteria* (0.2%).

**Figure 2:**
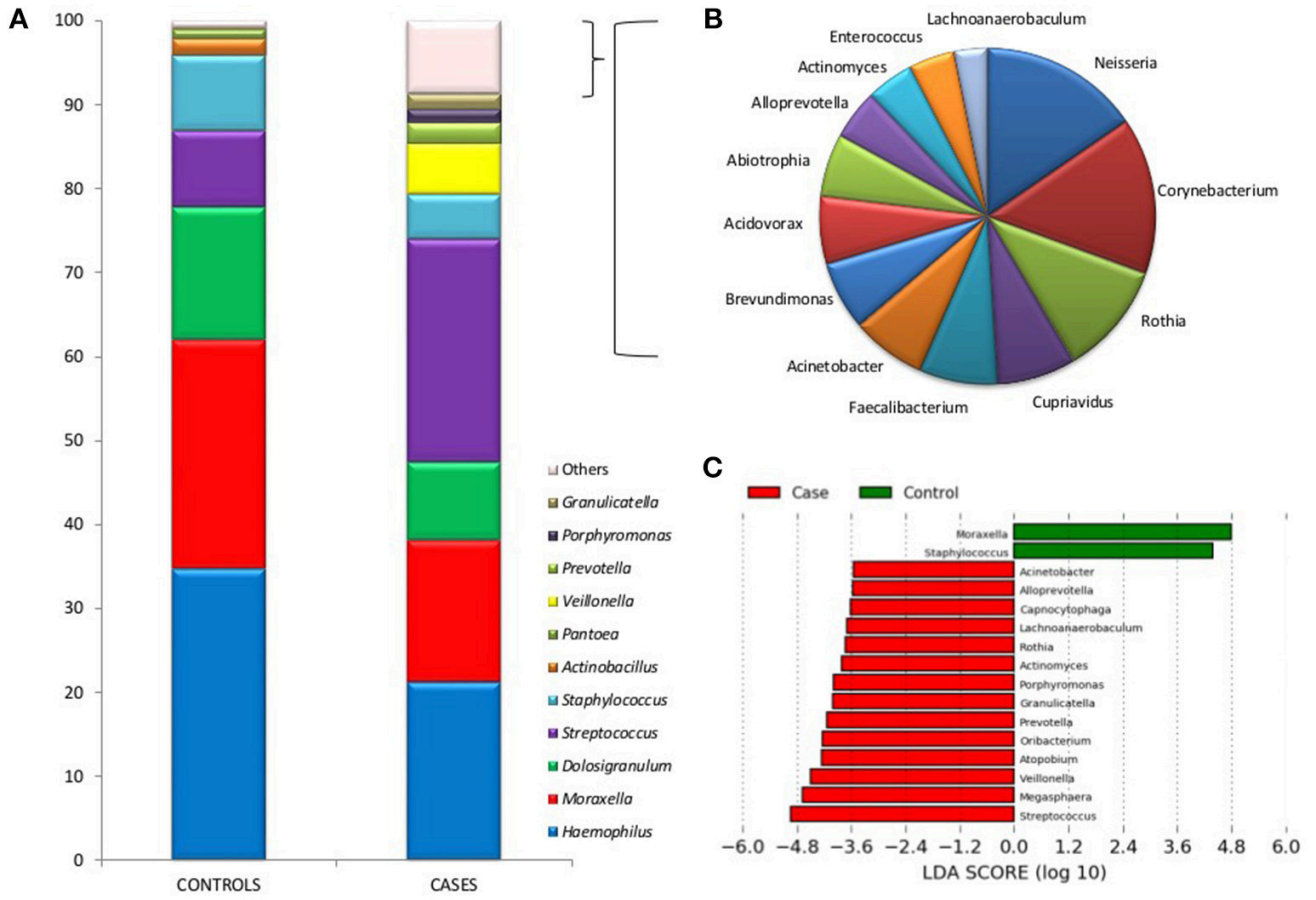
Bacterial taxonomic composition of nasopharyngeal samples in cases and controls. **(A)** Graphs show the mean proportion of the most frequent bacterial genera as inferred by PCR amplification and pyrosequencing of the 16S rRNA gene. The mean proportions were calculated based on 1,000 sequences per sample. Those bacteria at a proportion lower than 1% are indicated as “Others” and were particularly abundant in cases. **(B)** Shows a description of those low-frequency bacteria in patients with IPD, which include many oral organisms (bacteria at a proportion <0.2% are not shown). **(C)** A Linear Discriminant Analysis Effect Size (LEfSe) analysis shows those bacteria with significantly different levels (α = 0.05) between cases and controls.

Figure 2A shows differences between the two groups in the mean proportions of each bacterial genera. In the cases group the most prevalent bacteria were *Streptococcus, Haemophilus, Moraxella, Dolosigranulum, Veillonella, and Staphylococcus* with 26.4, 21.1, 16.8, 9.3, 6.1, and 5.2% of the total, respectively. Whilst in the control group their proportions were 9.0, 34.5, 27.1, 15.7, 0.1, and 8.9%, respectively. The higher bacterial diversity detected in children with IPD corresponded to the high prevalence of lactate fermenting *Veillonella*, and mainly to genera found at low frequencies. The taxonomic assignment of those genera present at <1% of the total, which accounted for over 9% of the reads in IPD patients, is shown in Figure 2B and reveals the presence of many oral species like *Corynebacterium, Neisseria, Actinomyces*, or *Rothia*, among others.

LEfSE ranking analysis shows that *Moraxella* and *Staphylococcus* were found to be significantly more abundant in healthy individuals, according to this high-dimensional class comparison test. On the other hand, the genera *Streptococcus, Megasphaera, Veillonella, Atopobium, Oribacterium, Prevotella, Granulicatella, Porphyromonas, Actinomyces, Rothia, Lachnoanaerobaculum, Capnocytophaga, Alloprevotella*, and *Acinetobacter* were significantly more abundant in patients with IPD (Figure 2C). Most of these bacteria are common oral inhabitants (Mira et al., 2017). Supplementary Figure 2 shows a Heatmap profile showing abundances of bacterial genera in individual cases and controls indicating the age of each child.

The use of long-amplicon (780 bp) sequencing allowed us to taxonomically assign the 16S sequences at the species level with a higher degree of accuracy (Shin et al., 2016). In order to minimize errors in taxonomic assignment, only sequence alignments >97% sequence identity over >500 bp were considered, which would encompass the 16S rRNA hypervariable regions v1–v3/v4. Our results are shown in Table 2. According to this assignment, the genus *Staphylococcus* would be dominated by *S. aureus* in the controls (>40% of the reads in this genus) whereas this species would be totally absent in IPD patients, that would be dominated by *S. epidermidis* and *S. haemolyticus*. Virtually, all *Haemophilus* species in controls would correspond to *H. influenzae*, whereas in the cases, 18% of the reads in this genus would correspond to uncultured or unknown species. It is surprising to note that over 90% of the reads matching *Streptococcus* would correspond to *S. pneumoniae* in the controls while in IPD patients, only 51% of the streptococcal reads would correspond to *S. pneumonia*e and over 40% of them gave top matches to *S. mitis, S. oralis*, or uncultured species.

**Table 2:** Bacterial species identified in the present study.

**Genus**	**Species**	**Cases (*n* = 28)**	**Controls (*n* = 28)**
*Streptococcus*	*pneumoniae*	51.08%	91.17%
	*mitis*	23.10%	2.82%
	*oralis*	7.02%	0.51%
	sp	6.62%	1.07%
	uncultured sp	5.40%	1.49%
	*infantis*	3.19%	0.13%
	sp. oral clone	1.84%	0.64%
	*peroris*	0.74%	–
	*sanguinis*	0.41%	0.77%
	*parasanguinis*	0.22%	–
	*genomosp*	0.12%	–
	*pseudopneumoniae*	0.10%	–
	*cristatus*	0.07%	0.47%
	*pyogenes*	0.02%	0.94%
*Dolosigranulum*	*pigrum*	83.26%	84.17%
	uncultured sp	16.74%	15.83%
*Haemophilus*	*influenzae*	80.30%	99,65%
	uncultured sp	11.77%	0,08%
	sp	6.81%	0,03%
	*parainfluenzae*	1.02%	0,09%
	*haemolyticus*	0.11%	0,10%
*Moraxella*	*catarrhalis*	94.89%	79.71%
	*lincolnii*	2.93%	8.30%
	*nonliquefaciens*	2.14%	4.46%
	*caprae*	0.04%	0.40%
	*lacunata*	–	7.14%
*Prevotella*	uncultured sp	34.54%	60.00%
	*histicola*	15.70%	–
	sp	12.80%	20.00%
	sp. oral clone	12.32%	–
	*pallens*	9.90%	–
	*nanceiensis*	3.86%	20.00%
	*conceptionens*	3.38%	–
	*oris*	2.90%	–
	*melaninogenic*	1.69%	–
	*salivae*	0.72%	–
*Veillonella*	uncultured sp	39,94%	24.00%
	*parvula*	29,49%	24.00%
	sp	14,23%	8.00%
	*dispar*	7,37%	24.00%
	sp. oral taxon	6,67%	20.00%
	*tobetsuensis*	2,24%	–
*Porphyromonas*	uncultured sp	71.93%	56.25%
	sp. *oral clone*	14.91%	18,75%
	sp	13.16%	25.00%
*Staphylococcus*	*epidermidis*	65,40%	9.67%
	sp	11,16%	47.12%
	*haemolyticus*	7,07%	0.05%
	uncultured sp	6,51%	2.23%
	*pettenkoferi*	3,35%	–
	*devriesei*	2,51%	–
	*cohnii*	1,95%	–
	*hominis subsp. null*	1,40%	–
	*pasteuri*	0,28%	–
	*warneri*	0,28%	–
	*aureus*	–	40.89%

*Data indicate percentages of 16S rRNA reads from a given genus with a hit against each species. Frequencies of each genera are shown in Figure 2. To minimize taxonomic assignment errors, only hits with >97% nucleotide identity and >500 bp alignment were selected. Only species with frequencies >0.1% are shown*.

### Microbiota Nasopharyngeal-Types in Health and Disease

**Figure 3:**
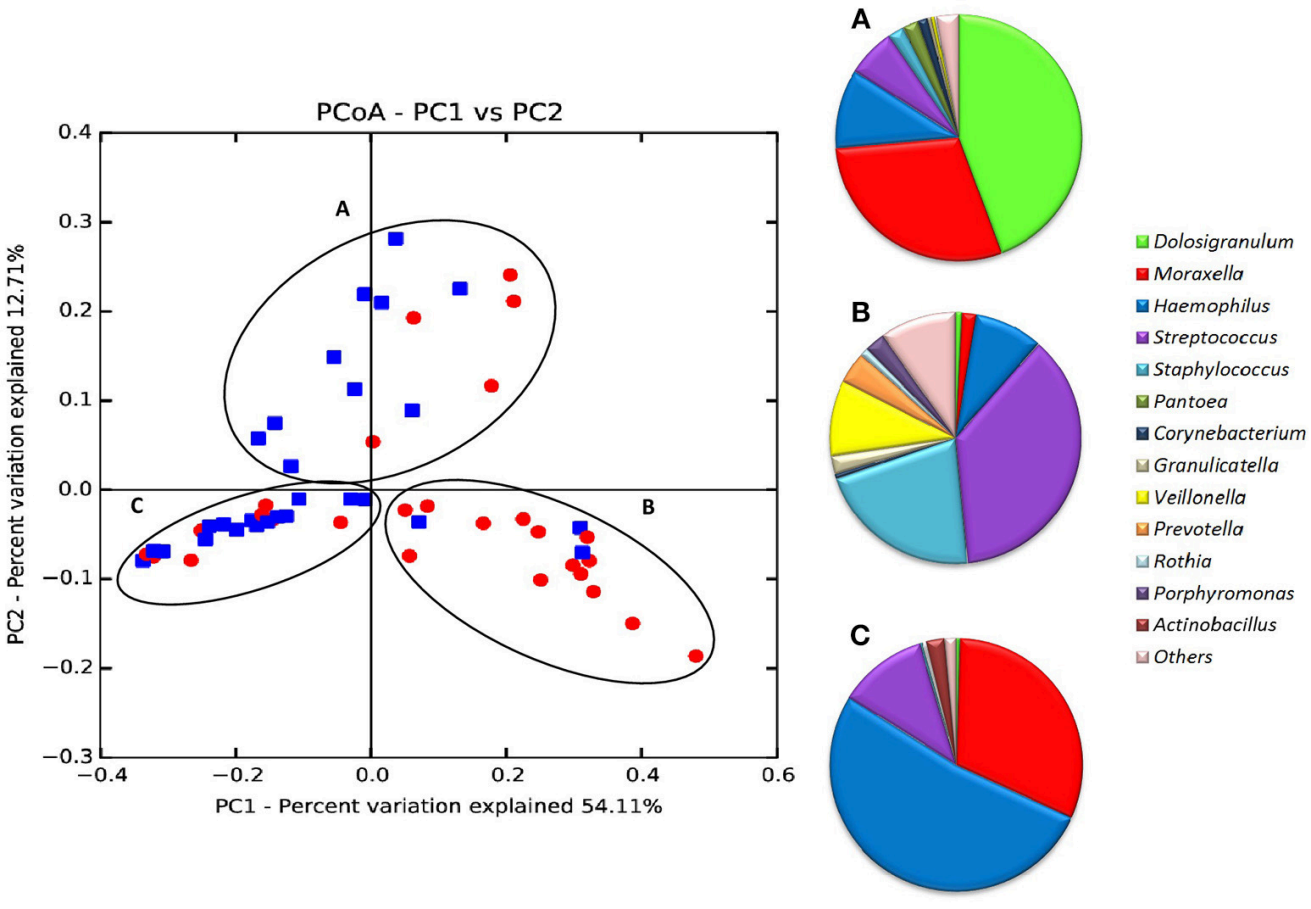
Principal Coordinates Analysis (PCoA) of all 56 nasopharyngeal samples according to bacterial composition. Data include IPD patients (red-dots) and healthy controls (blue dots). PCoAs were performed with weighted UniFrac analysis with clustering at the species taxonomic level (97% sequence identity) with 1,000 reads per sample. The taxonomic composition (proportion of bacterial genera) of the 3 microbiota types (“nasopharyngeal-types”) is shown to the right. Data in **(A–C)** were obtained from 1,000 randomly selected sequences per sample.

**Figure 4:**
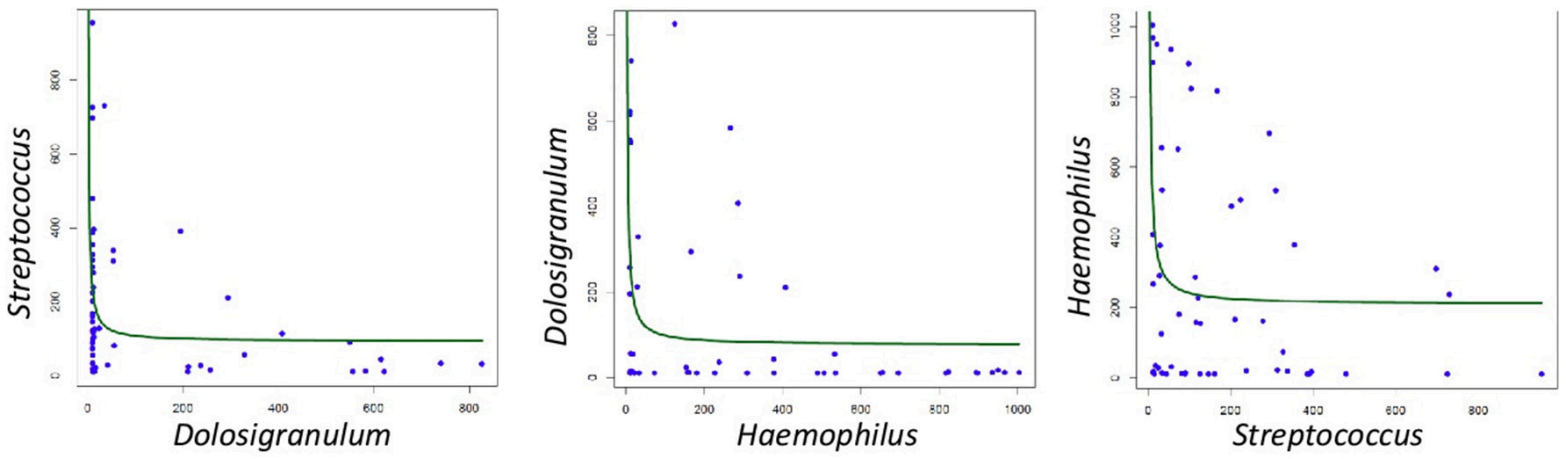
Correlations between the frequency of dominant bacteria in the three nasopharyngeal microbiota types. Data show potential negative associations, as they followed significant hyperbolic regressions.

When all samples were analyzed by two-dimensional principal coordinates analysis (2D PCoA), 67% of the variability in the data could be explained by the first two components. Samples appeared to cluster in three different groups, corresponding to three different types of respiratory tract microbiota, or nasopharyngeal-types (Figure 3). The Microbiota A was mainly composed of reads assigned to the genera *Dolosigranulum* (44.3%), *Moraxella* (29.3%), and *Haemophilus* (10.5%) (Figure 3A). The Microbiota B was represented mostly by the genera *Streptococcus* (36.9%), *Staphylococcus* (21.3%), *Veillonella* (9.8%), together with a high diversity of anaerobic genera as *Prevotella* and *Porphyromonas* (Figure 3B). Finally, the Microbiota C was composed mainly of the genera *Haemophilus* (52.1%), *Moraxella* (31.4%), and *Streptococcus* (11.4%) (Figure 3C). Thus, nasopharyngeal-type A was dominated by *Dolosigranulum*, nasopharyngeal-type C by *Haemophilus*, and nasopharyngeal-type B by *Streptococcus*, with a high presence of oral microorganisms. The three microbiota types were found to be significantly different from each other (Unifrac Distance, corrected *p* < 0.01 in all cases). In addition, CCA analysis showed that microbiota type significantly explained the variability in bacterial composition among samples (Adonis *p*-value: 0.002). In agreement with the existence of specific bacterial communities dominated by a given genus, negative correlations were found (Figure 4) between the three dominant genera (*p-*values for significant hyperbolic regressions were, respectively, 0.029, 0.011, and 0.029 for comparisons between *Dolosigranulum* and *Streptococcus, Dolosigranulum* and *Haemophilus*, and *Streptococus* and *Haemophilus*).

In order to understand the features influencing nasopharyngeal-types, all available epidemiological, microbiological, immunological and clinical variables were compared by bivariate analysis with the three microbiota types. No significant differences were found in any of the variables considered that could explain the grouping in the three different microbiotas (Table 3). However, the classification of patients into case and control groups was significantly associated with the nasopharyngeal-types (*p* = 0.006, Chi-square test). Among cases, nasopharyngeal-type B was the most frequently detected pattern (50.0%), followed by nasopharyngeal-type C (32.1%), while nasopharyngeal-type A was detected only in 17.9% of children with IPD. Conversely, nasopharyngeal-type B was only detected in 3 controls (10.7%).

**Table 3:** Epidemiological, microbiological, immunological, and clinical characteristics by microbiota type.

**A**				
**Variable**	**Microbiota A (*n* = 15)**	**Microbiota B (*n* = 17)**	**Microbiota C (*n* = 24)**	***p*-value**
Case group	5 (17.9)	14 (50.0)	9 (32.1)	**0.006**
**EPIDEMIOLOGICAL VARIABLES**				
Age, months (Md, IQR)	40.8 (20.8–80.3)	41.4 (32.1–56.6)	44.4 (29.5–61.4)	0.95
Gender, male	8 (53.3)	8 (47.1)	16 (66.7)	0.43
Influenza season	6 (40.0)	6 (35.3)	14 (58.3)	0.29
Race, Caucasian	11 (73.3)	16 (94.1)	21 (87.5)	0.26
Birth weight, gr (Mean, SD)	3,147 (689)	3,329 (749)	3,198 (428)	0.68
Gestational age, weeks (Mean, SD)	38.1 (2.3)	38.6 (2.6)	39.7 (1.7)	0.43
Delivery type, C-section^†^ (*n* = 48)	6 (42.9)	4 (28.6)	7 (30.4)	0.75
Breastfeeding	13 (86.7)	13 (76.5)	16 (66.7)	0.43
Breastfeeding in controls	9 (90.0)	1 (33.3)	9 (60.0)	0.10
Breastfeeding duration ≥ 6 months^¶^ ^†^(A: *n* = 13, B: *n* = 13, C: *n* = 16)	7 (53.9)	7 (53.9)	8 (50.0)	0.97
Breastfeeding duration, months (Md, IQR)^†^ (A: *n* = 13, B: *n* = 13, C: *n* = 16)	6 (3–10)	6 (3–12)	5.5 (1.5–7)	0.67
Day-care attendance	13 (86.7)	16 (94.1)	19 (79.2)	0.40
Shared bedroom^†^ (B: *n* = 12, C: *n* = 21)	5 (33.3)	7 (58.3)	13 (61.9)	0.21
Parental smoking^†^ (B: *n* = 15)	8 (53.3)	6 (40.0)	10 (41.7)	0.71
Vaccinated against influenza	0 (0.0)	0 (0.0)	1 (4.2)	1.00
Vaccinated against pneumococcal disease	9 (60.0)	13 (46.5)	14 (58.3)	0.45
PCV13 vaccinated	7 (77.8)	11 (84.6)	13 (92.9)	0.61
Respiratory infection in the previous 30 days^†^ (*n* = 52)	6 (42.9)	5 (31.3)	15 (68.2)	0.07^*^
**MICROBIOLOGICAL VARIABLES**				
Sp detection in NP sample by PCR	7 (46.6)	9 (52.9)	18 (75.0)	0.16
High-attack rate Sp serotype in NP sample	3 (42.9)	3 (33.3)	6 (33.3)	0.90
PCV13 serotype coverage in NP sample	3 (42.9)	4 (44.4)	6 (33.3)	0.81
Viral detection in NP sample by PCR	7 (46.7)	8 (47.1)	16 (66.7)	0.34
Multiple respiratory viral infection	2 (28.6)	3 (37.5)	4 (25.0)	0.82
**IMMUNOLOGICAL VARIABLES (CASE GROUP)**				
White blood count, 10^3^cells/mm^3^^†^(*n* = 21) (Md, IQR)	24.6 (18.9–33.4)	15.7 (10.0–18.2)	18.2 (7.5–22.7)	0.12
C-reactive protein, mg/L^†^ (*n* = 20) (Md, IQR)	29.2 (10.8–69.0)	113.1 (50.5–237.6)	302.9 (261.5–355.8)	0.009^**^
**B**				
**Variable**	**Microbiota A (*****n*** **=** **5)**	**Microbiota B (*****n*** **=** **14)**	**Microbiota C (*****n*** **=** **9)**	***p*****-value**
**CLINICAL VARIABLES (CASE GROUP)**				
Underlying disease	1 (20.0)	1 (71.4)	1 (11.1)	0.75
Antibiotic intake in the previous 7 days^†^ (*n* = 26)	5 (100.0)	12 (85.7)	6 (66.7)	1.00
Lenght of hospital stay, days (Md, IQR)	4 (3–6)	7 (7–8)	7.5 (3.5–10)	0.21
Admission to PICU	0 (0.0)	4 (28.6)	1 (11.1)	**0.53**
Lenght of PICU stay, days (Md, IQR)	0 (0.0)	2 (0–6)	1 (1–1)	0.45
Complications	0 (0.0)	2 (14.3)	2 (22.2)	0.80
**MAIN CLINICAL DIAGNOSIS**				
Complicated pneumonia	0 (0.0)	8 (57.1)	5 (55.6)	0.09
Bacteremia	3 (60.0)	3 (21.4)	1 (11.1)	0.17
Pneumonia	2 (40.0)	2 (14.3)	2 (22.2)	0.51
Meningitis	0 (0.0)	1 (7.1)	0 (0.0)	1.00
Sepsis	0 (0.0)	0 (0.0)	1 (11.1)	0.50
Serious IPD (complicated pneumonia, meningitis, or sepsis)	0 (0.0)	9 (64.9)	6 (66.7)	**0.04**
Course of disease, sequelae or exitus	0 (0.0)	3 (21.4)	2 (22.2)	0.67

*Results expressed as counts (%), unless stated otherwise*.

*Md, median; IQR, Interquartile range; SD, standard deviation; NP, nasopharyngeal; PCV13, 13-valent pneumococcal conjugate vaccine; PICU, Pediatric Intensive Care Unit; IPD, Invasive Pneumococcal Disease*.

† *Missing values*.

¶ *According to WHO recommendations*.

* *p = 0.05 comparing Microbiota B and C*.

** *p = 0.03 comparing Microbiota A and C; p = 0.04 comparing Microbiota A and B. The bold values means statistically significant associations with p-values < 0.05*.

Overall, children with nasopharyngeal-type A showed to have markedly lower inflammatory activity measured by the C-reactive protein level when compared to those with nasopharyngeal-type C (*p* = 0.03) or nasopharyngeal-type B (*p* = 0.04). In addition, no case with nasopharyngeal-type A was diagnosed with complicated pneumonia, meningitis, and sepsis, in contrast to the relatively high occurrence of these serious IPD manifestations among nasopharyngeal-type B and nasopharyngeal-type C cases (0.0, 64.9, and 66.7%, respectively, *p* = 0.04). Similarly, none of the cases with nasopharyngeal-type A required intensive care but a noticeable proportion of cases with nasopharyngeal-type B and C did (0.0, 28.6, and 11.1%, respectively). Of note, 90% of the ten healthy controls with a Microbiota A were fed with maternal milk whereas breastfeeding was less frequent in controls with Microbiota B (33.3%) and C-types (60.0%). In spite of these differences, the association between breastfeeding and type of microbiota was not found to be statistically significant. On the other hand, there were no significant correlations between any specific bacterial genera over-represented in IPD patients and inflammatory parameters.

## Discussion

The current work describes for the first time the nasopharyngeal microbiota in a case-control study of children with IPD and healthy children. Our data show that bacterial richness and diversity were significantly higher in IPD patients. In such cases, a clear dysbiosis was observed with a high frequency of *Veillonella* and other oral microorganisms which appeared to be relatively absent in controls. This over-representation of anaerobic and proteolytic oral species in children with IPD was also found by Segal et al. (2013) in adult bronchoalveolar lavage samples in association with increased inflammation. In our samples, the microbiota related with IPD was also associated with higher levels of the C-reactive protein inflammatory biomarker.

De Steenhuijsen Piters et al. (2016) similarly found higher nasopharyngeal microbiota diversity in elderly pneumonia patients compared to elderly healthy controls, whilst this difference was not found in adult patients. In ecological terms, these results are surprising since higher bacterial diversity is usually related with health (Turnbaugh et al., 2007), while lower bacterial diversity is associated with disease. It is noteworthy that patients with primary immunodeficiencies presented a higher microbiota diversity compared with healthy controls. This fact might correlate with an increase of immune system permissiveness for microbe colonization (Oh et al., 2013). Similarly, a local increase in bacterial diversity has been detected in polyps and tumor biopsies from patients with colorectal cancer (Mira-Pascual et al., 2015), a disease which has been associated with immune suppression at the affected tissues. The best studied case of immune-driven dysbiosis is probably gum disease, where the presence of the “keystone pathogen” *Porphyromonas gingivalis* has been shown to induce a profound alteration of the immune system (Hajishengallis, 2011) facilitating the settlement of many species that produce inflammation. However, the increase in microbial diversity associated with dysbiosis in gum disease and cancer could not only be due to immune alteration but also to a nutritionally richer environment (Mira et al., 2017). In the respiratory tract of children with IPD, future investigations should elucidate whether the observed dysbiosis is a consequence of immune changes, increased nutrient availability or both. In periodontal disease, destructive inflammation generates abundant gum tissue breakdown products that serve as nutrients for proteolytic and saccharolytic bacteria to obtain essential amino-acids and iron, including degraded collagen, and heme-containing compounds (Hajishengallis et al., 2012). It is important to underline that *Porphyromonas* was one of the over-represented genera in IPD patients (Figure 2C). However, all reads belonging to this genus appeared to correspond to uncultured or unknown species (Table 2). We hope the present study stimulates research into the characterization of these potentially pathogenic species, including their potential involvement as keystone pathogens (Darveau et al., 2012).

In addition, IPD samples presented significantly higher levels of *Veillonella* (Figure 2C), which is a bacterium that uses lactate as a carbon source, as a consequence of which it is usually found physically and functionally associated to lactate producers like *Streptococcus* (Dige et al., 2014; Gaspar et al., 2014). In our data, however, the correlation between *Streptococcus* and *Veillonella* levels in cases was not significant (R^2^ = 0.17, *p* > 0.1, Supplementary Figure 3) and therefore it is unknown whether streptococcal serotypes associated with IPD produce more lactate that those found in healthy children. Future work should determine whether the seemingly higher acidic environment in children with IPD is a consequence of lactate production by microorganisms, by lactate-dehydrogenase over-expression in the human tissue (Tan et al., 2002) or both.

Our data identified three bacterial clusters or nasopharyngeal-types, dominated by *Dolosigranulum, Streptococcus*, or *Haemophilus*. Similarly, other studies have described the presence of bacterial nasopharyngeal clusters. In a Danish cohort (1–3 months of age) and a Dutch cohort (from birth to 6 months of age), up to 5 and 4 nasopharyngeal-types were described (Mortensen et al., 2016), respectively. Although there are methodological, geographical and age-related differences between these studies, all available data from respiratory tract samples point toward the presence of precise microbial communities with a given dominant bacteria on each nasopharyngeal-type, although larger samples sizes are required to validate this.

In the current manuscript, the only statistically significant explanatory factor for these three microbiota profiles was the classification of patients into case or control groups. *Streptococcus*-dominated community (Microbiota B) was clearly associated to IPD patients. Given that *Dolosigranulum* abundance in the nasopharynx has been reported to be inversely associated with episodes of wheezing and mild respiratory tract infections (Biesbroek et al., 2014) we speculate that the *Dolosigranulum*-dominated community (Microbiota A) could be more resistant to pneumococcal infection occurrence and severity. Our hypothesis was supported by the lower number of cases with a Microbiota A-type (17.9% of the total) and by the fact that such cases experienced less severe manifestations of IPD and did not require intensive care. In this respect, it is suggestive that *Dolosigranulum* and *Haemophilus* are found in the present study to have a negative correlation with *Streptococcus*, the dominant genera in IPD cases (Figure 4). Moreover, the nasopharyngeal presence of *Dolosigranulum* has been associated with breastfeeding. It has been shown that lactation has a profound impact in the microbial community composition of the infant upper respiratory tract, increasing the prevalence and levels of *Dolosigranulum* and reducing the levels of *Staphylococcus, Prevotella*, or *Veillonella* (Biesbroek et al., 2014). In agreement with this, we found a considerably higher proportion of Microbiota A healthy controls that were breastfed and a trend for statistical significance in the relation between exclusive breastfeeding up to 6 months of age and case-control classification (*p* = 0.06) (Table 1). Despite the lack of statistical power to observe significance in these associations, the results obtained suggest that breastfeeding could have an important role in protection against pneumococcal infection. As far as for *Haemophilus*-dominated community (microbiota C), both IPD cases and healthy controls appear to fall within this microbiota profile. This genus, together with *Moraxella*, has been associated with higher rates of parent-reported upper respiratory infections and wheezing in the first years of life (Hyde et al., 2014; Bogaert et al., 2015). In fact, the group of children with *Haemophilus* and *Moraxella-*dominated microbiota showed a higher percentage of patients presenting a respiratory infection in the previous 30 days before the sample collection (68.2%), with a trend for significance (*p* = 0.07). However, future experimental work with a larger sample size should further explore the significance of these microbiota profiles and test whether the presence of a given nasopharyngeal microbiota makes individuals more sensitive or resistant to pneumococcal infection.

Our study describes the use of a long-amplicon sequencing approach of the 16S rRNA for species-level identification. Computer simulations show that taxonomic assignment accuracy, especially at the species level, decreases dramatically in short reads, like those of current Illumina or Ion Torrent technologies (Claesson et al., 2010), while the use of long-amplicon (≥500 bp) has been postulated as a good tool for species-level taxonomical assignments (Shin et al., 2016) and has been widely used as a diagnostic tool of bacterial infections in clinical samples (Guembe et al., 2013; Martínez et al., 2016). However, we have found surprising results using 16S rRNA for species level identification since *S. pneumoniae* would be more frequent in controls than in cases. These results are discordant with the results generated from a real-time PCR targeting *LytA* gene, the target recommended by CDC for pneumococcal detection. *LytA* PCR indicated the presence of *S. pneumoniae* in 64.4% of cases vs. 57.1% in controls (Table 1). According to our results, taxonomic assignment by using 16S rRNA could be limited in some genera and accurate identification at the species level should be done with more conserved genes. It is important to keep in mind that streptococci are particularly similar in the 16S rRNA gene sequence, and therefore species-level assignment in this genus must be treated with caution (Ing et al., 2012). Even more, despite *LytA* is the recommended gene utilized for pneumococcal diagnosis according to CDC (National Center for Immunization and Respiratory Diseases, 2014), this target has been reported to misidentify the pathogen (Morales et al., 2015; Simões et al., 2016), may be showing the occurrence of genetic exchange among streptococcal species and opening the possibility that other etiological agents could be involved. Thus, future work should increase our understanding of these microbial consortia by accurate species-level identification.

## Conclusion

In conclusion, we found a higher bacterial diversity and richness in children with IPD which could suggest an impaired immune response. This lack of immune competence could be aggravated by limited breastfeeding lower than recommended by WHO and by the presence of keystone pathogens which need to be characterized and that could favor the overgrowth of many proteolytic anaerobic organisms from the oral cavity, giving rise to a dramatic dysbiosis. From an applied point of view, we found suggestive microbiota profiles associated to IPD (*Streptococcus*-dominated microbiota profile) or asymptomatic colonization (*Dolosigranulum*-dominated microbiota profile) that could be used, respectively, as disease biomarkers or to identify health-associated inhabitants of the respiratory tract. The characterization of beneficial bacteria could be useful to prevent pneumococcal infections by integrating those microorganisms in a probiotic formula. The present study suggests not only respiratory tract samples, but also breast milk, as a potential source of those beneficial bacteria.

## Data Availability Statement

Sequence files and metadata for all samples used in this study are stored in the MG-RAST server to be publicly available by accessing the project Invasive Pneumococcal Disease (IPD) in children is associated with a highly diverse nasopharyngeal microbiota: a case-control study, ID mgp80930. All remaining data are contained within the paper and/or Supporting Information Files.

## Author Contributions

AM and CM-A contributed to the conception, design, and analysis of data. DH and JR contributed to the case and control recruitment and microbiological characterization of samples. AC-C and AG contributed to the bioinformatics analysis. PB contributed to the coordination, statistical analysis, and data/sample management. AC-C, PB, AM, and CM-A were the major contributors in writing the manuscript. All authors read and approved the final manuscript.

### Conflict of Interest Statement

The authors declare that the research was conducted in the absence of any commercial or financial relationships that could be construed as a potential conflict of interest.

## Supplementary Material

Supplementary Figure 1 Rarefaction curves of individual nasopharyngeal samples in **(a)** healthy children (controls) and **(b)** patients with invasive pneumococcal disease (cases). The horizontal axis shows the number of reads (sequencing effort) obtained by pyrosequencing the 16S rRNA gene. The vertical axis shows the number of operational taxonomic units (OTUs) at a level of 97% (estimated number of bacterial species per sample).

Supplementary Figure 2 Heatmap profile showing average abundances of bacterial genera incases and controls indicating the age of each child. Genera present in <3 patients were removed from the analysis for clarity.

Supplementary Figure 3 Relationships between the levels of *Streptococcus* and *Veillonella* in children's nasopharyngeal samples. Scatterplots show the number of sequence reads in the two bacterial genera for cases **(A)** and controls **(B)**.
