# Supplementary figures and images for: Nasopharyngeal Microbiota in Children With Invasive Pneumococcal Disease: Identification of Bacteria With Potential Disease-Promoting and Protective Effects
Source: Front Microbiol. 2019 Jan 28;10:11. doi: 10.3389/fmicb.2019.00011 (PMC6360994; doi:10.3389/fmicb.2019.00011)

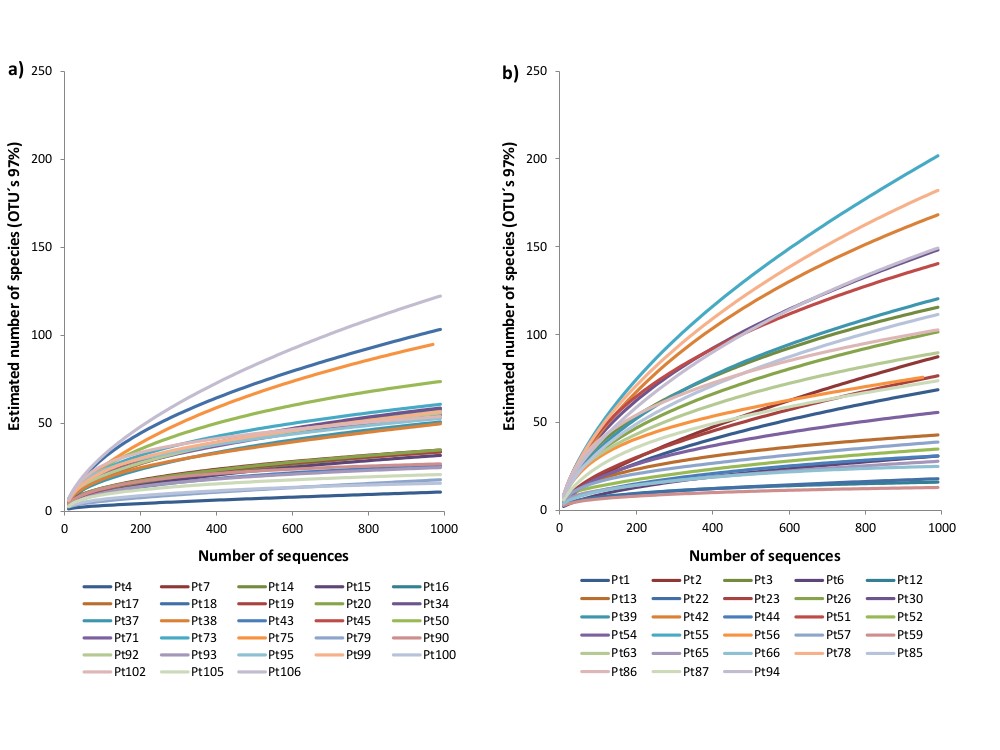

Supplement: Supplementary Figure 1 — Rarefaction curves of individual nasopharyngeal samples in (a) healthy children (controls) and (b) patients with invasive pneumococcal disease (cases). The horizontal axis shows the number of reads (sequencing effort) obtained by pyrosequencing the 16S rRNA gene. The vertical axis shows the number of operational taxonomic units (OTUs) at a level of 97% (estimated number of bacterial species per sample). [file Image_1.JPEG]

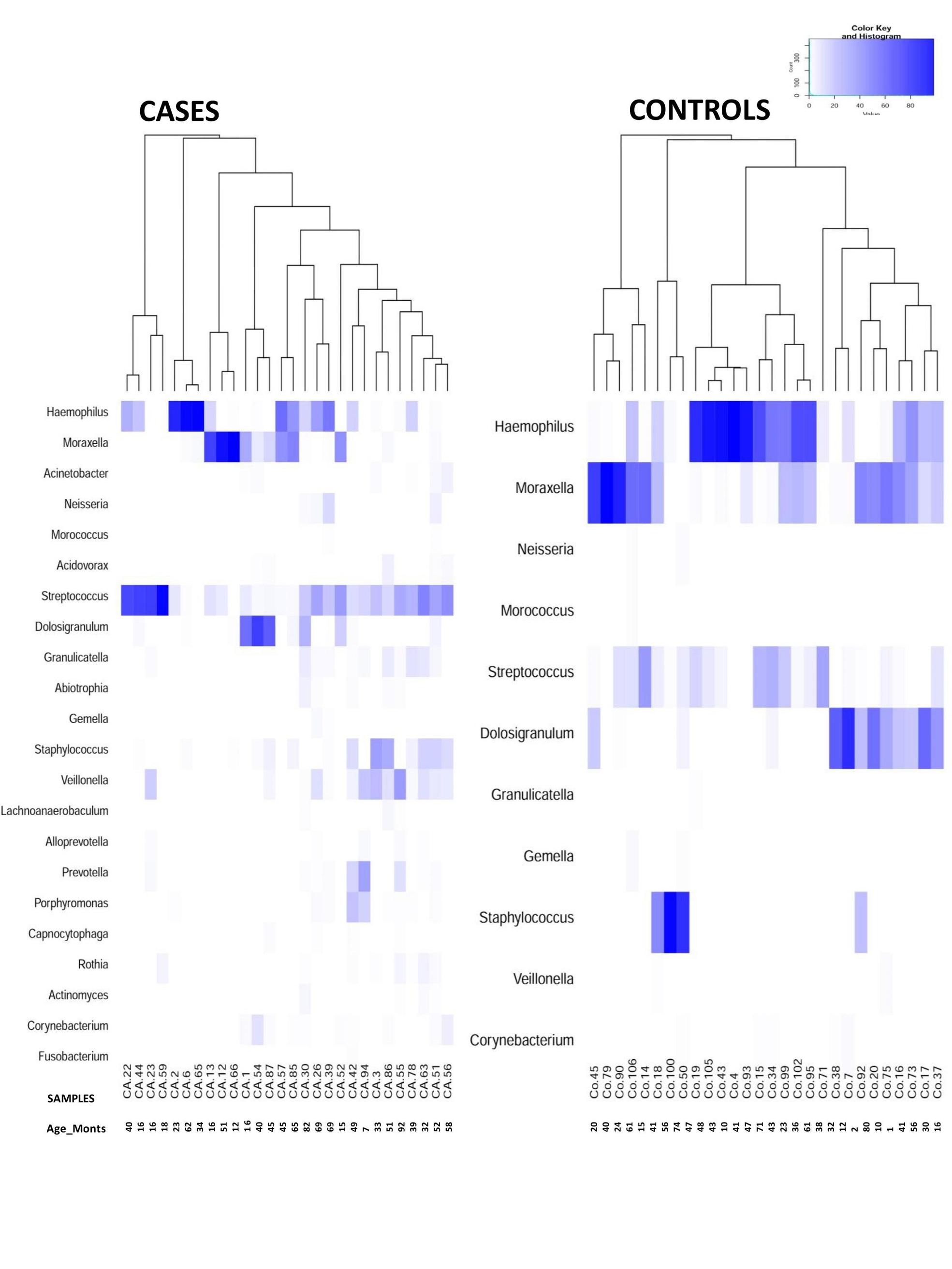

Supplement: Supplementary Figure 2 — Heatmap profile showing average abundances of bacterial genera incases and controls indicating the age of each child. Genera present in <3 patients were removed from the analysis for clarity. [file Image_2.JPEG]

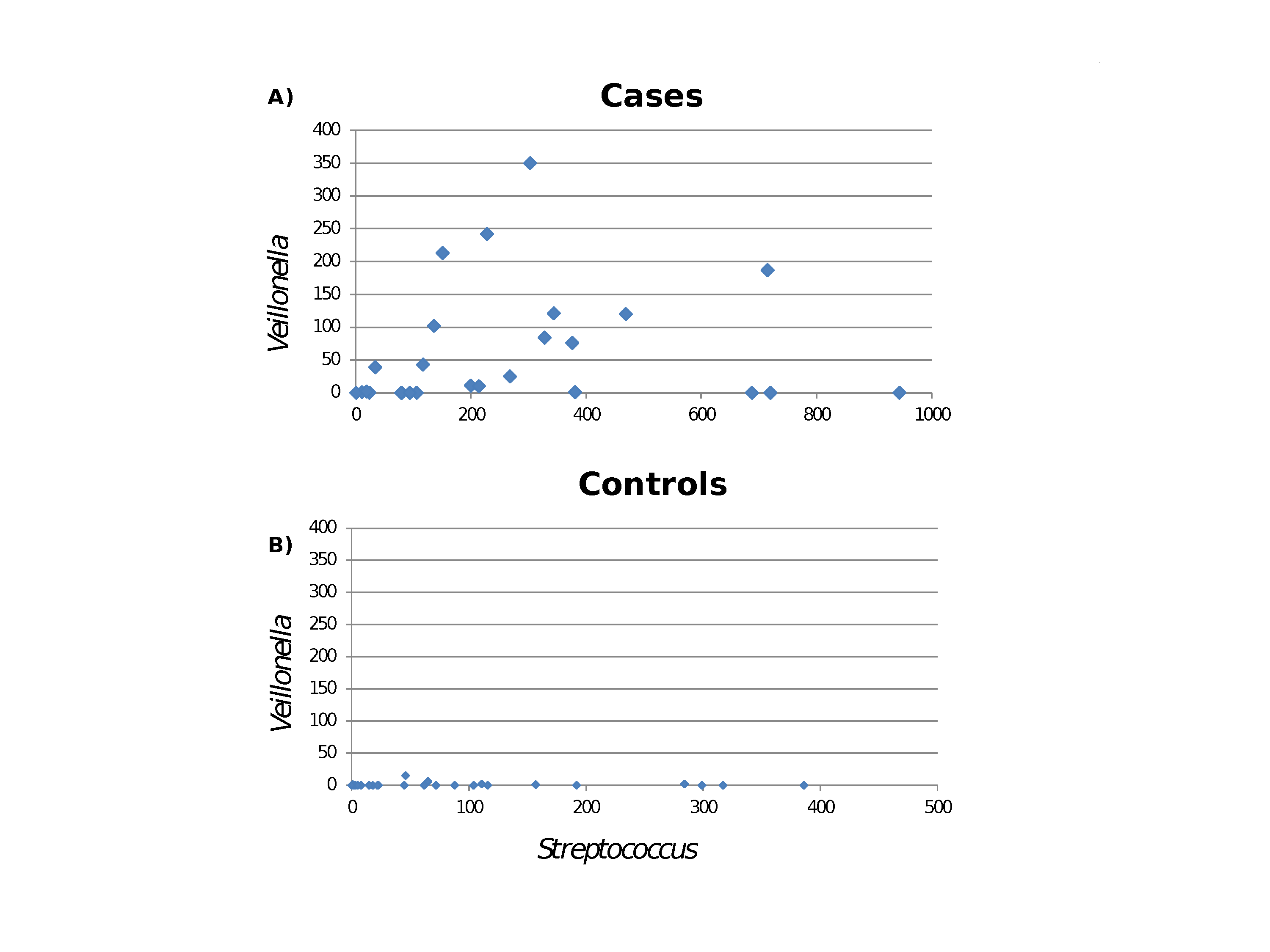

Supplement: Supplementary Figure 3 — Relationships between the levels of Streptococcus and Veillonella in children's nasopharyngeal samples. Scatterplots show the number of sequence reads in the two bacterial genera for cases (A) and controls (B). [file Image_3.TIFF]
